# A Novel In‐Cell ELISA With Superior Sensitivity and Specificity for the Detection of African Swine Fever Virus‐Specific IgM and IgG Antibodies

**DOI:** 10.1155/tbed/6272844

**Published:** 2026-01-05

**Authors:** Ping Wu, Aric J. McDaniel, Yelitza Y. Rodríguez, Vivian O’Donnell, Wei Jia

**Affiliations:** ^1^ Foreign Animal Disease Diagnostic Laboratory, National Veterinary Services Laboratories, Animal and Plant Health Inspection Service, United States Department of Agriculture, Plum Island Animal Disease Center, 40550 Route 25, Orient Point, New York, 11957, USA, usda.gov

## Abstract

African swine fever (ASF), a high‐profile transboundary animal disease caused by ASF virus (ASFV), imposes a devastating impact on the global swine industry. Given that vaccines are still under development, including field evaluations, early detection of ASFV is crucial for effective disease control and mitigation. Although PCR is the primary viral detection method of acute or subacute ASFV infections, antibody detection plays a unique role in detecting low‐virulent ASFV infection, identifying recovered animals, and tracking viral transmission. ELISA for ASFV antibody detection is commonly used for initial serological screening. To avoid false positive results, the World Organisation for Animal Health (WOAH) recommends using a second serologic method, such as the indirect immunofluorescence assay (IFA), indirect immunoperoxidase test (IPT), or immunoblot test, to confirm the ELISA‐positive cases. This strategy improves specificity but not sensitivity (i.e., false negative cases persist). To address this issue, a novel in‐cell ELISA (icELISA) was developed in this study. Receiver operating curve analysis of the icELISA revealed the optimized cutoff value of sample‐to‐positive ratio (S/P ratio) was at 47% with 99.46% analytical sensitivity and 99.43% analytical specificity. Results of the comparative diagnostic sensitivity analysis showed that positive detections of icELISA (150 samples) surpassed a blocking ELISA‐IPT combination (132 samples) by 18 samples. Further investigation revealed that the 18 samples contained ASFV‐specific immunoglobulin M (IgM) antibodies instead of immunoglobulin G (IgG). The results suggested the icELISA can detect both ASFV‐specific IgG and IgM, which outperforms a blocking ELISA‐IPT combination in earlier detection, particularly when only IgM antibody is present in a test sample.

## 1. Introduction

African swine fever (ASF), caused by ASF virus (ASFV), is a highly contagious and often fatal viral disease affecting all breeds of swine (*Sus scrofa*), including wild boars and domestic pigs. First described in Kenya in 1921 [1], ASF has since become endemic in sub‐Saharan Africa. Following its introduction to the Caucasus region of Georgia in 2007, the current outbreak rapidly spread across Eastern, Central, and Western Europe [2]. In 2018, ASF was introduced to China [3], leading to further dissemination throughout East and Southeast Asia. In 2021, ASF reached the Dominican Republic and Haiti [4]. This global spread has made ASF the most significant threat to the global swine industry.

Depending on the virulence of virus, ASF clinical conditions may vary from peracute, acute, subacute, and chronic to subclinical [5–8]. Infected suids in all but peracute cases typically produce antibodies against ASFV that persist for months or even years after the virus has cleared [9]. While ASFV PCR is the primary method for viral DNA detection in acute or subacute cases, serological detection of ASFV antibodies also plays a unique role in detecting low‐virulent ASFV infection, identifying recovered animals, and tracking viral transmission [10]. ELISAs are commonly used for rapid screening of large numbers of samples for ASFV antibodies; however, the World Organisation for Animal Health (WOAH) recommends that ELISA‐positive results should be confirmed with a second serological method such as an indirect immunofluorescence assay (IFA), indirect immunoperoxidase test (IPT), or immunoblot test [9] to avoid false positive results. The confirmation test does not aim to address the potential false negative issue, and the issue is often overlooked.

Antibodies are immunoglobulin (Ig) proteins produced by a host’s immune system in response to an infection. Immunoglobulin M (IgM) and immunoglobulin G (IgG) are two major antibody classes induced by ASFV infection. IgM can be detected as early as 3 days after infection and provides the first line of antibodies in immune defense, after which high‐affinity IgG responses are initiated [11]. Thus, IgM could be one of the major antibody classes for early detection by immunoassays. Selection of broad‐spectrum antigen targeting by both IgM and IgG is critical when developing an ELISA designed for high sensitivity and early detection. Common ELISAs for ASFV antibody detection, such as those in commercial kits, typically utilize one or a limited number of ASFV proteins [12, 13] or epitopes [14], such as p30 [15, 16], p54 [17, 18], p72 [19], p62 [20], p17 [21], pB602L [22], K205R [23, 24], CD2v [18, 25], I329L [26], p11.5 [27], p22 [12], or p15 [28]. Limiting the antigenic spectrum to one or two proteins may potentially reduce sensitivity due to fewer antibody binding sites. Gallardo’s findings suggested that a blocking ELISA using the single viral protein p72 as antigen for ASFV antibody detection may have resulted in false‐negative results [29]. Reis et al. [30] demonstrated that the pig’s initial immune response may not be triggered by dominant viral proteins like p30, p54, and p72, which suggested that ELISAs based on these proteins could also lead to a false‐negative result when a test sample was collected in the early stages of the disease. In comparison, using infected cells as antigen, IPT has exhibited high sensitivity and early detection [9]; however, IPT is a microscope‐based method which is labor‐intensive and demands highly skilled staff for both test execution and accurate result interpretation. To address the drawbacks of ELISA and IPT, a cell‐based indirect ELISA (in‐cell ELISA or icELISA) was developed and evaluated for sensitivity, early detection, and high‐throughput potential in detection of ASFV antibodies in this study. Procedures for icELISA have been developed for cell function analysis by using specific monoclonal antibodies as detection agents to measure the target antigens in cells [31–33]. Several icELISA protocols are also used for detection of the autoantibodies in heart failure patients [34], SARS‐CoV‐2 neutralizing antibody [35], and rabies lyssavirus neutralizing antibody [36]. In this report, using ASFV‐infected cells as the antigen combined with a background cancelation strategy, we describe a novel, simple, quick, and automation‐capable icELISA with superior sensitivity and specificity, which can detect IgM and IgG against ASFV at the early stages of the disease in infected pigs.

## 2. Materials and Methods

### 2.1. Cell and Virus

SK6 cells, a swine kidney cell line, were cultured in Dulbecco’s Modified Eagle Medium (DMEM, ThermoFisher Scientific, Cat. No. 11995065) supplemented with 10% fetal bovine serum (FBS Millipore Sigma, Cat. No. F3885). ASFV Uganda 64 (10^7.9^ TCID_50_/mL) is a genotype X strain (GenBank: PP750552.1) which was adapted to swine cell line PK15 for 86 passages, then SK6 for 6 passages.

### 2.2. ASFV Antigen Plate Preparation and Optimization

Actively growing SK6 cells were harvested through trypsinization and suspended in DMEM supplemented with 10% FBS at a concentration of 7 × 10^5^ cells/mL. The cell suspension was then seeded into a 96‐well tissue culture plate at 100 μL/well. The cells were incubated at 37°C in a 5% CO_2_ atmosphere for 24 h. After removing the media, 100 μL/well of a virus suspension (ASFV Uganda 64 strain diluted in DMEM supplemented with 4% FBS) was added. For the cell control wells, 100 μL/well of DMEM supplemented with 4% FBS (without virus) was added. The plate was then incubated under the same conditions for an additional 48 h. At the end of the incubation period, the supernatant was decanted, the monolayers were fixed with either formaldehyde or acetone/methanol, and the antigen plate was stored at −70°C.

#### 2.2.1. Determination of Fixing Methods

The ASFV antigen plates were prepared using SK6 cells infected with ASFV Uganda at 0.2 multiplicity of infection (MOI) and incubated for 48 h at 37°C in a 5% CO_2_ atmosphere. The antigen plates were fixed with 4% formaldehyde (Lab Alley, Cat. No. C3970) (F1) following the Abcam in‐cell ELISA protocol (https://docs.abcam.com/pdf/kits/in-cell-elisa.pdf) or by using 200 μL/well of acetone (30%) and methanol mixture (70%) (F2) for 8–10 min at room temperature [37]. The effect of the fixing methods on icELISA was assessed by using five sera from naïve pigs (Neg AS) and five sera positive for ASFV antibodies (Pos AS). These sera were diluted at 1:40 in blocking buffer.

#### 2.2.2. MOI and Duration of Infection

To optimize MOI and duration of incubation, ASFV Uganda 64 was diluted at 1:100, 200, 400, and 800 in DMEM supplemented with 4% FBS. The SK6 cell monolayers were infected with 100 μL of the diluted virus suspension with corresponding MOI of 0.4, 0.2, 0.1, 0.05, and 0.03. The infected plate(s) were incubated at 37°C in a 5% CO_2_ atmosphere and fixed at 24‐, 48‐, and 72‐h postinoculation with an acetone (30%) and methanol (70%) mixture (stored at 4–8°C) for 8 min at room temperature. The effects of MOI and duration of infection on icELISA were assessed by using a hyperimmune serum against ASFV at a 1:20,000 dilution.

#### 2.2.3. Blocking Buffer Selection

Two formulations of blocking buffers were evaluated. Buffer 1 (B1) was PBS‐T (0.5 mL of Tween 20 (Millipore Sigma, Cat. No. P1379) in 1000 mL of 1 × PBS (ThermoFisher Scientific (Cat. No. 70013‐032), pH 7.2) with 2.1% NaCl and 10% KPL milk Diluent/Blocking solution concentrate (Seracare, Cat. No. 5140‐0011); and Buffer 2 (B2) was PBS‐T with 2.1% NaCl, 15% calf serum (Cytiva, Cat. No. SH30087.02), and 5% normal goat serum (Millipore Sigma, Cat. No. G9023). The antigen plates fixed with acetone/methanol were blocked with either B1 or B2 at 37°C for 60 min. The effects of blocking buffer on icELISA results were assessed by using 10 Neg AS and 10 Pos AS at a 1:40 dilution.

### 2.3. Procedure of the icELISA for ASFV Antibody Detection

The 96‐well antigen plates, both ASFV‐infected and cell control wells, were blocked by using 120 μL/well blocking buffer at 37°C for 60 min or overnight at 4°C. After decanting the blocking buffer, 100 µL of freshly prepared sample and negative control (diluted at 1:40 in blocking buffer) and appropriately diluted positive control were added to the ASFV‐infected wells and cell control wells in duplicate. The plate was incubated at 37°C for 60 min, followed by four washes with PBS‐T. Goat anti‐pig IgG (Fc)‐HRP conjugate (ThermoFisher Scientific, Cat. No. PAI‐84628) and goat anti‐pig IgM‐HRP conjugate (Bio‐Rad, Cat. No. AA148P) were diluted in blocking buffer at 1:10,000 and 1:20,000, respectively. The mixed conjugate was added (100 μL/well) to the plate and the plate was incubated at 37°C for 45 min. The plate was washed 4 times with PBS‐T, then 100 μL of 1‐step ultra‐TMB ELISA substrate (ThermoFisher Scientific, Cat. No. 34028) was added to each well, followed by 10 min of incubation at room temperature for color development. The reaction was stopped by adding 100 μL/well of 1N H_2_SO_4_ solution. Optical density (OD_450_) was measured within 5 min after stopping the reaction. The net OD_450_ value (ODnet) of each sample was calculated using the following formula:

ODnet=Average OD of ASFV wells−average OD of cell control wells .

### 2.4. Blocking ELISA (bELISA)

A bELISA for detection of ASFV antibody was performed using a commercial ELISA kit, Ingezim PPA COMPAC ELISA kit 11.PPA.k3 (Gold Standard Diagnostics), which utilizes the ASFV p72 protein as capture antigen and its monoclonal antibody as the detection antibody. The bELISA was validated and routinely used for detecting ASFV antibodies at the Foreign Animal Disease Diagnostic Laboratory (FADDL).

### 2.5. IPT

IPT was conducted in accordance with the protocol previously reported [37] which was validated and routinely used as a confirmatory test for ASFV antibodies at FADDL. Samples were diluted 1:40 in blocking buffer and tested in duplicate, and the results were read with an inverted optical microscope. Intense red cytoplasmic precipitation was interpreted as ASFV antibody positive. The absence of red precipitation was interpreted as negative for ASFV antibodies.

### 2.6. Kinetic Analysis of Host IgM and IgG Response to ASFV Challenge

To characterize the dynamic humoral immune response following ASFV exposure, a kinetic analysis of host IgM and IgG antibodies was performed. Six independent sets of time‐course serum samples were acquired from Dr. Chung. Samples were collected at seven designated time points: 0, 4, 8, 10, 12, 14, and 21 days post‐exposure (dpe). The onset of viremia in the source animals was previously confirmed by ASFV PCR to be between 8 and 10 dpe [38]. The anti‐ASFV IgM and IgG antibodies in the serum were subsequently detected using icELISA.

### 2.7. Indirect IFA

SK6 cells at a concentration of 1 × 10^5^ cells/mL were seeded into each well of 8‐chamber slides (0.3 mL/well) and incubated at 37°C in 5% CO_2_ atmosphere for 24–48 h to form an 80%–90% confluent monolayer. ASFV Uganda 64 was diluted in DMEM supplemented with 4% FBS at a concentration of 4 × 10^4^ pfu/mL. The monolayer of SK6 cells was infected with 100 μL of the diluted virus suspension (0.1 MOI) and incubated under the same conditions for another 24 h. The infected monolayers were fixed with acetone/methanol (30/70%) at room temperature for 8 min. The IFA was performed according to WOAH guidance [9], with samples diluted 1:40 and goat anti‐pig IgM‐FITC (Bio‐Rad, Cat No. AAI48F) and goat anti‐pig IgG (Fc)‐FITC (Bio‐Rad, Cat. No. AAI41F) each diluted 1:500 in PBS‐T/2.1% NaCl. The slides were read with a ZOE Fluorescent Cell Imager microscope (Bio‐Rad).

### 2.8. Analytical Sensitivity, Specificity, and Cutoff Point

The analytical sensitivity and specificity of the icELISA were evaluated using a total of 292 serum samples, including 106 ASFV‐positive sera and 186 sera from naïve pigs. The ASFV antibody status of these samples was prescreened by blocking ELISA and confirmed by IPT. The samples were diluted 1:40 in blocking buffer, and the positive control was diluted 1:40,000 and negative control 1:40 in the same buffer. The sample‐to‐positive ratio (S/P ratio) was calculated using the following formula:

Sample/positive ratio %=100×sample ODnetpositive ODnet.

The S/P ratios were analyzed using the receiver operating characteristics (ROC) analysis to determine the accuracy of icELISA [39]. The cutoff value of the S/P ratio and the analytical sensitivity and specificity were determined using Square of Distance and Youden Index methods.

### 2.9. Preliminary Repeatability and Limit of Detection (LOD)

Three batches (batches 1, 2, and 3) of ASFV‐infected antigen plates were used to evaluate well‐to‐well consistency (repeatability) and batch‐to‐batch variation (reproducibility) of icELISA. The initial assessment of reproducibility was conducted using a serially diluted hyperimmune serum against ASFV (IPT titer between 1:64,000 and 128,000 dilution) in three independent tests. LOD of the hyperimmune serum in icELISA was determined based on the average ODnet value from the three independent tests. Preliminary repeatability of the icELISA was conducted using a panel of 14 ASFV antibody‐positive sera at a 1:40 dilution. The test was repeated five times: two times with batch 1, one time with batch 2, and two times with batch 3. The results were evaluated using single‐factor ANOVA based on the S/P ratio.

### 2.10. Diagnostic Sensitivity and Specificity of icELISA

A total of 361 serum samples were used for the diagnostic sensitivity and specificity analysis, including 104 samples collected by FADDL from experimentally infected pigs and 223 samples collected by FADDL from recent ASF outbreaks in the Caribbean and African regions. An additional 34 domestic serum samples were used to evaluate the cross‐reaction of icELISA and were obtained from the National Veterinary Services Laboratories and Kansas State University consisting of 6 sera against classical swine fever virus (CSFV), 6 sera against foot‐and‐mouth disease virus (FMDV), 6 sera against vesicular exanthema of swine virus (VESV), 1 serum against porcine epidemic diarrhea virus (PEDV), 1 serum against swine influenza A virus (SIV), 7 sera against porcine reproductive and respiratory syndrome virus (PRRSV), 1 serum against swine pox virus (SPV), 1 serum against senecavirus A (SVA), and 5 sera against porcine circovirus 2 (PCV‐2). The samples were diluted 1:40 in blocking buffer, and each sample was tested in duplicate. The S/P ratio of each sample was calculated, and the established cutoff value of the S/P ratio in Section 2.8 was used to determine the ASFV antibody status for each sample. These samples were also tested using bELISA and IPT in accordance with WOAH guidelines.

### 2.11. Data Analysis

The accuracy of icELISA was evaluated using ROC and area under the curve (AUC) analysis. Binomial 95% confidence interval (95% CI) was estimated using the Clopper–Pearson method. Method comparisons were analyzed using ANOVA and Tukey–Kramer test (for pairwise comparison) or chi‐square test.

## 3. Results

### 3.1. Optimization of the icELISA

Determination of fixation method: The average ODnets of five Neg AS were consistently below 0.1 on the ASFV antigen fixed with F1 or F2. Single‐factor ANOVA analysis indicated no significant difference (*p* > 0.05) between F1 and F2 on negative samples. The average ODnet of five Pos AS on the ASFV antigen fixed with F2 was 0.5 which was significantly higher than 0.08 on the ASFV antigen fixed with F1 (*p* < 0.01) (Figure 1a).

Figure 1Optimization of the icELISA. (a) Fixing methods: the ASFV‐infected plate was fixed with 4% formaldehyde (F1) or 30% acetone/70% methanol (F2). The icELISA results indicated the average net optical density (ODnet) of 5 naïve serum samples (Neg AS) was lower than 0.1, and no significant difference was found between F1 and F2 (*p* > 0.05). The average ODnet of 5 ASFV antibody positive samples (Pos AS) with F2 was significantly higher than that with F1 (*p* < 0.01). (b) Blocking methods: PBS‐T/2.1% NaCl/10% KPL milk (B1) or PBS‐T/2.1% NaCl/15% calf serum/5% goat serum (B2) was evaluated in icELISA. The average ODnet of 10 Neg AS was lower than 0.1, and no significant difference was found between B1 and B2; the average ODnet of 10 Pos AS with B2 was significantly higher than that with B1 (*p* < 0.01). (c) MOI/infection duration: plots of ODnet of icELISA with respect to incubation time and MOI. The ASFV‐infected plates were prepared with MOI of 0.4, 0.2, 0.1, 0.05, and 0.03 of Uganda 64 strain. The infection was stopped at 24, 48, and 72 h postinfection. The ELISA results indicated that the ODnet value was significantly increased when increasing the incubation time from 24 to 72 h (*p* < 0.01) at all MOI levels. The ODnet values were also significantly increased when increasing the MOI values (*p* < 0.01).

**Figure:**
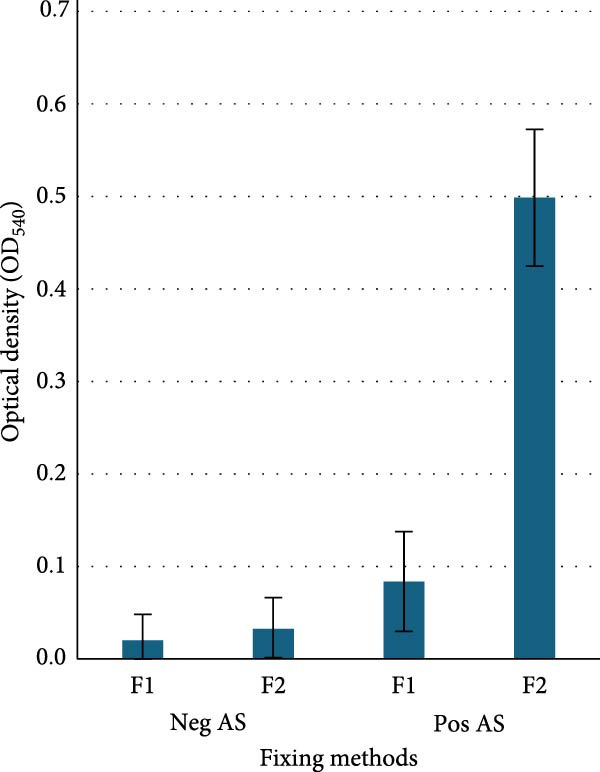
(a)

**Figure:**
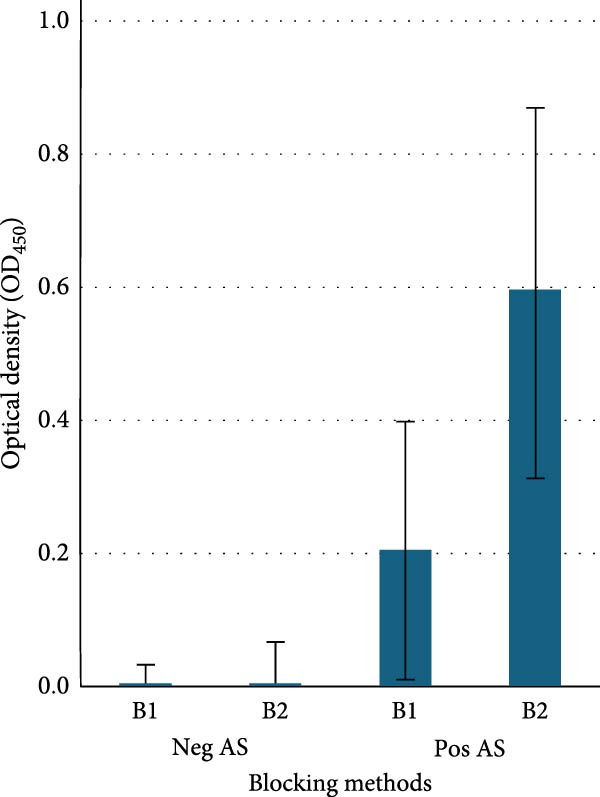
(b)

**Figure:**
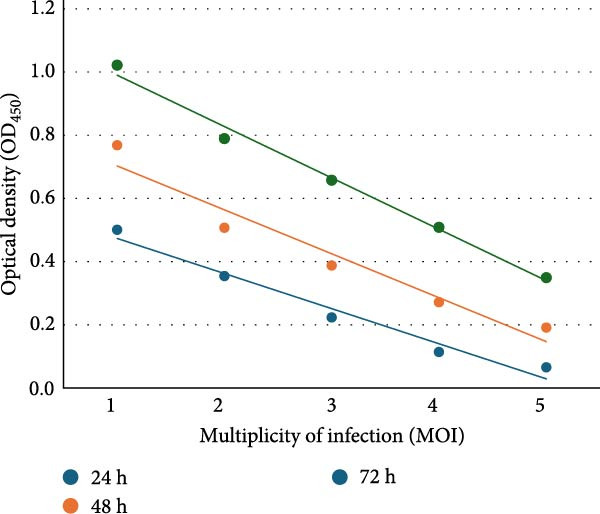
(c)

Blocking buffers: The average ODnets of Neg AS between B1 and B2 were also consistently below 0.1. Single‐factor ANOVA analysis indicated no significant difference between B1 and B2 on negative samples (*p* > 0.05). Conversely, the average ODnet of Pos AS with B2 was 0.6, which was significantly higher than 0.2 with B1 (*p* < 0.01) (Figure 1b).

Effect of MOI and incubation time on the icELISA: The icELISA results of the ASFV antigen from different MOI and incubation times indicated a positive correlation of the average ODnets between MOI as well as infection durations (Figure 1c). Two‐factor ANOVA analysis revealed significant increases of ODnets when increasing incubation time from 24 to 72 h (*p* < 0.01) at all MOI levels. On the other hand, there were significant increases in average ODnets when increasing MOI for all incubation times (*p* < 0.01).

### 3.2. Analytical Sensitivity, Specificity, and Cutoff Point

**Figure 2:**
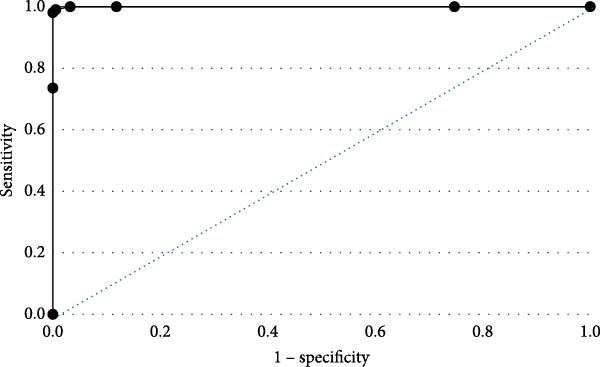
Plot of area under the curve (AUC). Receiver operating characteristics (ROC) analysis was conducted by using 186 naïve pig sera and 106 ASFV antibody‐positive sera (confirmed by indirect immunoperoxidase test). The ROC curve shows the accuracy value interpreted as AUC. The results indicated that the AUC was 99.98%, with a 95% confidence interval (95% CI) of 99.79%–100%.

The ROC analysis indicated the AUC, a measurement of icELISA accuracy, was 99.98% with 95% CI of 99.79%–100% (Figure 2). The optimized cutoff value of the S/P ratio was determined to be 47% by Square of Distance and Youden Index values. With the 47% cutoff, the analytical sensitivity and specificity were 99.46% (95% CI of 97.04–99.98%) and 99.43% (95% CI of 96.88%–100%), respectively. Adjusting the cutoff value to 50% would decrease sensitivity to 98.10% (95% CI of 93.35–99.77%), but the specificity would reach 100% (95% CI of 98.04%–100%). If the cutoff value were set to 44%, the sensitivity would be 100% (95% CI of 96.58%–100%), but the specificity would decline to 96.77% (95% CI of 93.1%–98.81%) (Table 1).

**Table 1:** The relationship between cutoff point of sample‐to‐positive ratio (S/P%), analytical sensitivity, and analytical specificity of the in‐cell ELISA (icELISA), assessed by receiver operating characteristic (ROC) analysis using 186 naïve pig sera and 106 ASFV antibody‐positive sera (confirmed by indirect immunoperoxidase test).

S/P ratio (%)	Analytical sensitivity		Analytical specificity		Square of distance/Youden index
	icELISA positive/total positive sera (%)	95% CI (%)	icELISA negative/total naïve sera (%)	95% CI (%)	
50	104/106 (98.10)	93.35–99.77	186/186 (100)	98.04–100	0.0004/0.9811
47	105/106 (99.46)	97.04–99.98	185/186 (99.43)	96.88–100	0.0001/0.9852
44	106/106 (100)	96.58–100	180/186 (97.16)	93.11–98.81	0.0010/0.9677

### 3.3. Preliminary Repeatability and LOD

**Figure 3:**
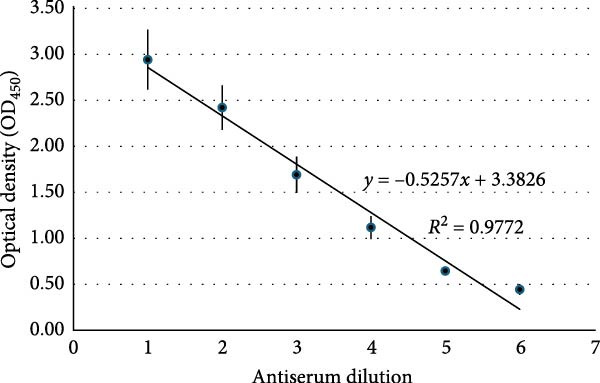
Plots of ODnet of icELISA with respect to limit of dilution (LOD) of a hyperimmune serum. LOD was evaluated using a hyperimmune serum against ASFV. The serum was 2‐fold diluted from 1:2500 to 1:320,000, and three tests were performed. The vertical lines indicate the standard deviation. Single‐factor ANOVA analysis indicated there was no significant difference between the 3 repeats (*p* > 0.05). The solid curve represents an average value of the 3 repeats and suggests the LOD of the hyperimmune serum in icELISA was between 1:65,000 and 1:130,000, which was comparable to the IPT titer (1:64,000–128,000).

The results of the initial reproducibility assessment indicated there was no significant difference (*p* > 0.05) among the three independent tests with three batches of ASFV antigen plates. The average ODnet values were 1.12 at a dilution of 1:20,000, 0.64 at 1:40,000, 0.44 at 1:80,000, and 0.21 at 1:160,000. The LOD of the hyperimmune serum was estimated between 1:65,000 and 1:130,000, which was comparable to the IPT titer. Based on these results, an ASFV antibody‐positive control of icELISA was established by diluting the hyperimmune serum to 1:20,000 to 40,000 with an estimated average ODnet value between 0.5 and 1.0 (Figure 3). The S/P ratios of five repeats for evaluation of preliminary repeatability are listed in Table 2. Single‐factor ANOVA analysis suggested that there were no significant differences (*p* > 0.05) between the five repeats.

**Table 2:** Preliminary repeatability of icELISA, assessed with a panel of 14 ASFV antibody‐positive sera.

Serum	Test 1	Test 2	Test 3	Test 4	Test 5	Average/SD
1	89	81	198	90	80	107/51
2	272	96	176	322	281	229/92
3	239	360	145	316	230	258/83
4	275	242	145	182	150	199/57
5	216	311	154	215	159	211/63
6	57	133	84	68	59	80/31
7	53	65	74	74	55	64/10
8	70	130	69	50	62	76/31
9	52	69	130	115	115	96/34
10	96	106	113	96	127	108/13
11	79	127	79	68	71	85/24
12	277	330	140	277	231	251/71
13	162	158	101	144	111	135/28
14	248	357	167	273	229	254/69

*Note*: The same set of samples was repeated five times at a 1:40 dilution. Sample‐to‐positive (S/P) ratios (%) were calculated, and the consistency of the icELISA was assessed by using single‐factor ANOVA. The results suggested that there was no significant difference (*p* > 0.05).

### 3.4. Diagnostic Sensitivity and Specificity of icELISA in Comparison With bELISA and IPT

Out of 104 experimental sera, bELISA detected 65 positives and 39 negatives for antibodies against ASFV. Of the 65 bELISA‐positive samples, 61 were confirmed positive by IPT (61/104, 58.65%), and the other 4 were deemed false positives; thus, there were 43 ASFV antibody negatives (43/104, 41.35%) by using the bELISA‐IPT protocol. In comparison, icELISA detected 74 antibody positives (74/104, 71.15%) and 30 antibody negatives (30/104, 28.85%) against ASFV based on the 47% cutoff. The 74 positives included all 61 positives from the bELISA‐IPT test and 13 more positives from the negative group of the bELISA‐IPT test (Table 3). Two of the four false‐positives samples by bELISA‐IPT were found to be IgM positive and the other two were negative by icELISA.

**Table 3:** Comparative diagnostic sensitivity and specificity of in‐cell ELISA (icELISA), blocking ELISA (bELISA), and indirect immunoperoxidase test (IPT) for ASFV antibody detection by using 3 sets of serum samples: (1) 104 samples collected from pigs experimentally infected with ASFV, (2) 223 field samples collected from regions with ASF outbreaks, and (3) 34 domestic swine antisera against major viral diseases other than ASF.

Sample	icELISA		bELISA		IPT	
	+	−	+	−	+	−
Experimental	74	0	61	0	61	0
Sample	0	30	4	39	0	43
Subtotal	74	30	65	39	61	43
Field sample	76	0	69	2	71	0
	0	147	3	149	0	152
Subtotal	76	147	72	151	71	152

Swine antisera against pig viral diseases other than ASF	0	34	0	34	0	34

Total	150	211	137	224	132	229

A total of 223 field samples were screened using bELISA; 72 samples tested positive and 151 were negative against ASFV. Only 69 out of 72 bELISA‐positive samples were confirmed by IPT. The 151 bELISA‐negative samples were also tested by IPT. The results indicated 149 out of 151 samples were negative, and 2 samples were positive against ASFV. The bELISA‐IPT tests resulted in a total of 71 positives (71/223, 31.84%) and 152 negatives (152/223, 68.16%). The bELISA‐IPT results also suggested three false positives (3/223, 1.3%) and two false negatives (2/223, 0.9%) from bELISA; these false positives and false negatives were also confirmed by icELISA. In comparison, out of 223 field samples, icELISA was able to identify 76 antibody positives (76/223, 34.08%) and 147 antibody negatives (147/223, 65.92%) against ASFV. The icELISA detected all 71 bELISA‐IPT positive samples and picked up five positives from the bELISA‐IPT negative samples (Table 3).

The 34 antisera against FMDV, CSFV, and the other major domestic swine viral diseases tested antibody negative against ASFV by either icELISA or bELISA and IPT (Table 3).

### 3.5. Kinetics of Antibody Response Following ASFV Challenge

**Figure 4:**
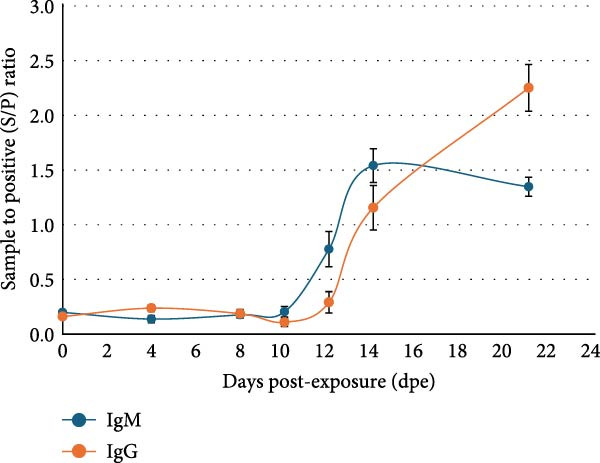
Plot of dynamic ranges of IgM and IgG response against ASFV challenge. The animals were exposed to ASFV contaminated environment at 0 dpe. Samples were collected at seven designated time points: 0, 4, 8, 10, 12, 14, and 21 dpe. The onset of viremia in the source animals was previously confirmed by ASFV PCR to be between 8 and 10 dpe. The samples were tested for IgM and IgG, respectively. Average sample‐to‐positive ratio was calculated from 6 sets of samples at each time point. The vertical lines indicate the standard error. The results indicated that the initial detection of IgM occurred at 12 dpe. The initial detection of IgG occurred at 14 dpe, which was 2 days after the initial IgM detection.

Across the 6 sets of time‐course serum samples, IgM seroconversion was first detected at 12 dpe by icELISA. The initial detection occurred 2–4 days after the earliest ASFV PCR positive in corresponding blood samples. The IgM titer reached its peak at 14 dpe, after which a decline was observed. The IgG response emerged slightly later, with seroconversion first detected at 14 dpe, which was 2 days after initial IgM positivity. Following the initial detection, the IgG titer demonstrated an increase from 14 dpe through the end of the observation at 21 dpe (Figure 4).

### 3.6. Indirect IFA

A total of 18 IPT‐negative but icELISA‐positive samples (13 experimental sera and 5 field sera) were tested for ASFV‐specific IgM and IgG antibodies by IFA. The results indicated 17 of the 18 samples were positive for IgM only, and 1 was positive for both IgM and IgG (Table 4).

**Table 4:** Detection of IgM and IgG in 18 samples positive by in‐cell ELISA (icELISA) but negative by indirect immunoperoxidase test (IPT) by using icELISA with only anti‐pig IgM‐HRP or IgG (Fc)‐HRP conjugate as the secondary antibody and indirect immunofluorescence assay (IFA) with anti‐pig IgM‐FITC or IgG (Fc)‐FITC conjugate as the detection antibody.

Serum sample	Days post infection (days post exposure)	IPT	icELISA		IFA	
			IgM‐HRP	IgG (Fc)‐HRP	IgM‐FITC	IgG (Fc)‐FITC
1	5	−	+	−	+	−
2	(17)	−	+	−	+	+
3	(13)	−	+	−	+	−
4	(16)	−	+	−	+	−
5	(16)	−	+	−	+	−
6	8	−	+	−	+	−
7	6	−	+	−	+	−
8	(14)	−	+	−	+	−
9	(12)	−	+	−	+	−
10	7	−	+	−	+	−
11	9	−	+	−	+	−
12	9	−	+	−	+	−
13	6	−	+	−	+	−
14	Field sample	−	+	−	+	−
15	Field sample	−	+	−	+	−
16	Field sample	−	+	−	+	−
17	Field sample	−	+	−	+	−
18	Field sample	−	+	−	+	−
Positive control (hyperimmune serum)		+	−	+	−	+
Negative control (naïve pig serum)		−	−	−	−	−

*Note*: The samples were tested at a 1:40 dilution.

## 4. Discussion

The icELISA for ASFV antibody detection utilizes ASFV‐infected cells as the capture antigen. The antigen spectrum of the infected cells is intricate, comprised of both viral antigens and cellular antigens. The latter may bind specifically or non‐specifically to antibodies, leading to a high background. To address the challenges posed by this antigenic complexity, we implemented several strategies, including optimization and a background‐canceling approach. Firstly, the choice of cell line was crucial. SK6 cells, a swine cell line, were selected over Vero cells because the background reaction of Vero cells was significantly higher than SK6 cells (Figure S1). Secondly, a background subtraction for ODnet was applied. ODnet was determined by subtracting the average OD of cell control from the average OD of ASFV‐infected cells.

There are two major types of fixatives: cross‐linking and precipitating. Formaldehyde‐based fixatives are the most common type of cross‐linking fixative used, and they work by covalently coupling molecules to each other, thus creating a stable meshwork within tissues that is not permeable to antibodies or intercellular proteins without additional treatment. Acetone and methanol act as precipitating fixatives that fix and preserve tissue integrity by breaking hydrogen bonds, removing water, precipitating proteins, and removing small soluble molecules and lipids. Additional permeabilization is not required for entry of larger protein molecules. Most icELISA protocols used paraformaldehyde to fix cell monolayers, followed by Triton X‐100 treatment for membrane permeabilization [31–35]. In this study, the effects of formaldehyde and acetone/methanol fixation on the ELISA ODnet were compared using both ASFV antibody‐negative and positive samples. Our results demonstrated that both fixation methods produced similar background ODnet values (less than 0.1), as evaluated by ASFV antibody‐negative samples. However, a significant improvement in the average ODnet of ASFV antibody‐positive samples was achieved with acetone/methanol fixation versus formaldehyde fixation (*p* < 0.01), suggesting that acetone/methanol is better for preserving ASFV antigens.

Impacts of MOI and duration of infection on icELISA results were evaluated in this study. The results indicated that lower MOI with shorter incubation times resulted in lower ODnet values. While extending the incubation time increased the ODnet, the background reaction was also increased. With a higher MOI, the cytopathic effect reached a high percentage too soon to form consistent monolayers, and extended incubation times with higher MOI eventually destroyed the monolayer of infected cells. In this study, we found that a 0.2 MOI infection for 48 h maintained the consistency of infected monolayers with an appropriate ASFV antigen expression profile.

The blocking step in an ELISA system is crucial for reducing background interference and improving the signal‐to‐noise ratio and sensitivity. Beyond its basic blocking function, the blocking buffer in the icELISA should effectively block primary and secondary antibodies from binding to molecules of the Ig superfamily (i.e., active blocking) on the cell surface. Blocking buffers formulated with skim milk and calf/goat serum were evaluated in this study. The results revealed that the average ODnet values with calf/goat formulated blocking buffer were almost three times higher than the skim milk formulated blocking buffer because of the reduced background reaction.

To assess the analytical sensitivity and specificity and establish a cutoff value for the S/P ratio, a total of 186 naïve pig sera and 106 ASFV antibody‐positive pig sera (confirmed by IPT) were tested by icELISA. The S/P ratio was calculated for each sample, and ROC analysis was used to evaluate the performance of the icELISA across various cutoff points of the S/P ratio. ROC results indicated a diagnostic accuracy (AUC) of 99.98% with a 95% CI of 99.79% to 100%. Square of distance and Youden index analyses indicated that a cutoff value at 47% of S/P ratio has an analytical sensitivity of 99.46% (95% CI of 97.04%–99.98%) and an analytical specificity of 99.43% (95% CI of 96.88%–100%). The cutoff value may be adjusted based on the intended purpose. For a confirmation test, a 50% cutoff value could be used to enhance specificity and reduce potential false positives. Conversely, a cutoff value at 44% could increase the sensitivity and reduce potential false negatives while sacrificing a small percentage of specificity; a 44% cutoff might be suitable for primary screening and surveillance testing. The analytical sensitivity was also assessed by evaluating LOD of a hyperimmune serum against ASFV. The results indicated that the LOD of the hyperimmune serum on icELISA was between the dilutions of 1:65,000 and 130,000.

The comparative diagnostic sensitivity and specificity between icELISA and bELISA with IPT were assessed by using a total of 361 serum samples from individual animals, including 104 experimental samples, 223 field samples, and 34 pig antisera against FMDV, CSFV, and several other major domestic swine viral diseases. Out of the 361 serum samples, icELISA detected 150 antibody positives against ASFV (41.55%) and 211 negatives (58.45%). In comparison, bELISA‐IPT tested 132 antibody positives against ASFV (36.57%) and 229 negatives (63.43%). The diagnostic specificity of icELISA was assessed by examining potential cross‐reactions with major swine viral diseases including CSFV, FMDV, VESV, PEDV, SIV, PRRSV, SPV, SVA, and PCV‐2. The icELISA results demonstrated no cross‐reaction with these diseases. Alternatively, icELISA showed broad inclusive detection against serum samples produced by using different ASFV strains such as Lisbon 60, Uganda 64, Malta 78, Georgia 2007, and DR2021 (Table S1).

Comparing icELISA and bELISA‐IPT, icELISA detected 18 more ASFV antibody‐positive samples than bELISA‐IPT, 13 of which were experimental samples and the other five were collected from the field. Further study of the 13 experimental samples indicated that they were collected from pigs at early stages of ASFV infection (5–9 days postinfection or 12–17 dpe) while IgM would be predominant in the host immune response. The kinetic analysis results also indicated that the initial IgM detection occurred as early as 12 dpe, which was 2–4 days following the onset of viremia in the source animals and at least 2 days earlier than IgG detection. It is possible, then, that the ASFV‐specific antibody in these 13 samples was dominated by IgM. In the IPT, the detection antibody is Protein A‐HRP conjugate, but Protein A does not naturally bind to pig IgM [40]. Therefore, if the samples were dominated by IgM, the IPT results would be expected to be negative.

ASFV is a large DNA virus with a complicated protein profile, and it is not clear which viral protein(s) trigger initial host immune response after ASFV infection. Prior research has indicated that the initial IgM response to ASFV infection is likely directed against the K205R protein rather than the more common proteins p30, p54, or p72 [30]. This implies that the bELISA targeting p72 antibodies may fail to detect early IgM responses. The icELISA uses whole viral antigens and both IgG (Fc) and IgM HRP conjugates that allow detection of IgM as well as IgG. To confirm this hypothesis, we cross‐examined the 18 icELISA‐only positive samples using a modified icELISA procedure where only anti‐pig IgM‐HRP conjugate or anti‐pig IgG (Fc)‐HRP conjugate was used. The results indicated that IgM and not IgG against ASFV were detected in these samples. The specific IgM against ASFV was also visualized by IFA with secondary antibody FITC conjugates specifically against pig IgM or IgG (Fc). The IFA results also indicated that specific IgM was detected in these 18 samples (Table 4). The current icELISA system cannot identify the specific viral protein(s) responsible for IgM binding, however. Further study to identify these proteins would not only significantly improve serological methods but also enhance our understanding of virus‐host interactions.

## 5. Conclusions

A novel icELISA was developed in this study using ASFV‐infected cells as the antigen to capture both IgM and IgG antibodies against ASFV. The analytical and diagnostic sensitivity and specificity of the method were partially validated. The icELISA showed superior sensitivity and specificity for detecting ASFV‐specific IgM and IgG antibodies at the early stages of the disease in infected pigs. In addition, the icELISA, like other ELISA‐based assays, has a high‐throughput potential for use as the primary method of large‐scale antibody detection.

## Disclosure

The findings and conclusions in this publication are those of the authors and should not be construed to represent any official U. S. Department of Agriculture or U.S. government determination or policy.

## Conflicts of Interest

The authors declare no conflicts of interest.

## Funding

No external funding was used to conduct this study.

## Supporting Information

Additional supporting information can be found online in the Supporting Information section.

## Supporting information


**Supporting Information** Figure S1: Nonspecific reactions of Vero and SK6 cells of icELSIA. The monolayers were fixed by acetone/methanol (30/70%). icELISA was performed with normal swine serum at 1:40 dilution. The conjugate (anti‐IgG‐HRP) was two‐fold diluted from 1:2560 (1), 5120 (2), 10240 (3), and 20480 (4). Table S1: Inclusivity of icELISA. The icELISA was performed with 14 various ASFV antibody‐positive samples at 1:40 dilution. The conjugate was anti‐IgG‐HRP and anti‐IgM‐HRP at 1:10,000 and 1:20,000, respectively.

## Data Availability

The data that support the findings of this study are available from the corresponding author upon reasonable request.
