# Supplementary material for: A Novel In‐Cell ELISA With Superior Sensitivity and Specificity for the Detection of African Swine Fever Virus‐Specific IgM and IgG Antibodies
Source: Transbound Emerg Dis. 2026 Jan 5;2026:6272844. doi: 10.1155/tbed/6272844 (PMC12766277; doi:10.1155/tbed/6272844)
Supplement: Supplementary file 1 — Supporting Information Figure S1: Nonspecific reactions of Vero and SK6 cells of icELSIA. The monolayers were fixed by acetone/methanol (30/70%). icELISA was performed with normal swine serum at 1:40 dilution. The conjugate (anti‐IgG‐HRP) was two‐fold diluted from 1:2560 (1), 5120 (2), 10240 (3), and 20480 (4). Table S1: Inclusivity of icELISA. The icELISA was performed with 14 various ASFV antibody‐positive samples at 1:40 dilution. The conjugate was anti‐IgG‐HRP and anti‐IgM‐HRP at 1:10,000 and 1:20,000, respectively. [file TBED-2026-6272844-s001.pdf]

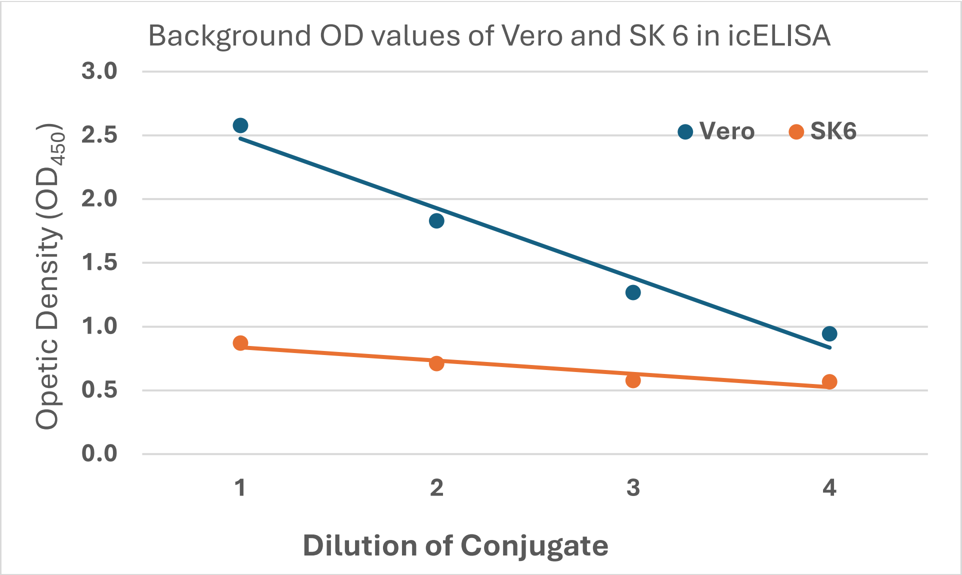

Fig 1. Non-specific reactions of Vero and SK6 cells of icELSIA. The monolayers were fixed by acetone/methanol (30/70%). icELISA was performed with normal swine serum at 1:40 dilution. The conjugate (anti-IgG-HRP) was 2-fold diluted from 1:2560 (1), 5120 (2), 10240 (3), and 20480 (4).

Table 1. Inclusivity of icELISA. The icELISA was performed with 14 various ASFV antibody positive at 1:40 dilution. The conjugate was anti-IgG-HRP and anti-IgM-HRP at 1:10,000 and 1:20,000, respectively.

	ASFV	DPI	icELISA	IPT	Ingenasa
1	Malta 78	21	+	+	+
2	Lisbon 60	43	+	+	+
3	Uganda 64	47	+	+	+
4	Georgia 2007	45	+	+	+
5	DR 2021	63	+	+	+
6	Brazil 78	56	+	+	+
7	Killean III	37	+	+	+
8	ET13/1505	93	+	+	+
9	Ourt88/3	97	+	+	+
10	NH/P68	72	+	+	+
11	Ourt88/3	97	+	+	+
12	E75	15	+	+	+
13	Ken05/Tk1	70	+	+	+
14	NH/P68+Arm07	126	+	+	+
